# Declaring Worldviews in SSM for Sustainability & Community Learning

**DOI:** 10.1007/s11213-025-09749-8

**Published:** 2026-01-08

**Authors:** Miles W. Weaver, Rebecca J. M. Herron, Kamila Pokorna, David E. Salinas Navarro, Eliseo Vilalta-Perdomo

**Affiliations:** 1 Centre for Business Innovations and Sustainable Solutions, Edinburgh Napier University, Edinburgh, UK; 2 University of Lincoln, Lincoln, UK; 3 Faculty of Mining and Geology, VSB-TUO, Ostrava, Czech Republic; 4 Universidad Panamericana, México City, México; 5 Aston University, Birmingham, UK

**Keywords:** Community OR, Learning, Soft Systems Methodology, Systems Thinking, Sustainability

## Abstract

For over fifty years, Soft Systems ideas and the Soft Systems Methodology (SSM) have played a pivotal role in understanding various problem situations and initiating action. Often tackling the grandest challenges of our time, SSM will retain continued relevance in helping decision-makers address sustainability challenges within organisations and their communities. In this paper, we are concerned with the *meaningful co-creation of sustainable value through community-based learning using SSM. *More specifically, recognising that a sustainability paradigm, characterised by the *need to create a just and safe space for humanity to thrive within the means of a living planet* (as called for by Raworth, 2017), is often marginalised or overlooked. This paradigm presents us with an ethical imperative, complex and messy challenges/issues, and a set of ideals (articulated in the United Nations Sustainable Development Goals) that are significantly off track. This paper employs a variation of the Delphi method, drawing on the authors’ collective interest and experience in applying SSM in communities, to propose a double-loop learning cycle to explore the underlying assumptions of our worldviews and mental models within communities. We suggest that an SSM learning cycle can be enhanced by initiating conversations on relevant *models for sustainability* (such as Doughnut Economics, UN SDGs, and the principles for a Circular Economy), to find common ground for triggering new learning. This idea is contextualised and proposed as the *value(s)-action gap* phenomenon, which can help explain the difference between an individual, an organisation, and/or a community's *intention(s)* and their actual *action(s).*In doing so, find common ground, shift to higher levels of systems consciousness from an ego-centric to an ecosystem level of awareness, engage communities, and take an intergenerational perspective. We suggest that incorporating a double-loop learning cycle into SSM can support organisations and their communities in putting shared values into meaningful action.

## Introduction

‘Soft’ systems thinking has evolved and been widely used for over 50 years to understand and improve real-world challenges in organisations and communities, where different stakeholders hold varying perspectives on the situation and potential actions. These ideas have been operationalised by applying *‘Soft Systems Methodology*,*’* an approach initially conceived by Checkland (1972) in its development (e.g., Checkland 1999, 2000; Checkland and Poulter 2006) and application. We now seek to revisit these ideas within the context of addressing grand sustainability challenges in communities. To do so, we will focus on SSM as a framework, which is part of the family of methods that spans *Soft ‘OR’/problem structuring* (PSMs), *Systems Thinking*, and *Community Operational Research* (See Mingers and Rosenhead 2004). SSM is one of many PSMs that have been adapted to address a wide range of business and community-based applications.

In this paper, we focus on what the authors argue is a key benefit of SSM: *supporting community learning through analysis that leads to meaningful action*. This goes to the heart of Community OR, which is defined as the “*meaningful engagement of communities”* (Midgley et al. 2018, p. 772). Although the notion of ‘community’ can be contested, we are concerned with collectives of people who share a common identity. This identity may be based on a common *geography* (such as a city or neighbourhood), *interest* (such as supporting a sports team), *economic* link (such as a discount club membership), or *social* connection (e.g., parents at a school). These collectives of people may (or may not) *cooperate* (e.g., signing up, paying a fee, agreeing to the rules) or *collaborate* (e.g., playing sports, volunteering for the club) around this shared identity. More widely, the term ‘stakeholder’ may be used to refer to those who have a direct or indirect interest in a decision, organisation, or community group. Their “stake” may be *financial* (such as an investor), *legal* (such as a regulatory body), *ethical* (such as an interest group) or *operational* (such as a supplier, customer, and employees). As this “stake” is easier to define and formalise, stakeholders are often considered and potentially involved in decision-making. In contrast, different communities are socially constructed more generally and informally; they are affected by an action but not necessarily involved (such as farmers’ markets). Here we make our distinction, in that SSM can help facilitate community learning by considering broader issues and interests that Midgley (1992) suggest can be treated as ‘sacred’ or ‘profane’.

Problem structuring has been widely suggested as necessary “up-front” in any OR intervention (Rosenhead and Mingers 2001). Weaver et al. (2025) call for engagement at higher levels of systems consciousness to address the diverse needs of different stakeholders and point out examples of SSM that have addressed sustainability challenges (e.g., Dijk et al. 2017; Weaver et al. 2018; Hadi et al. 2023; Núñez-Ríos et al. 2023). This concerns a need to consider broader community and stakeholder concerns as part of an OR/systems intervention. This is evident in a shift towards a sustainability paradigm, as opposed to adhering to the prevailing neoliberal paradigm. This has influenced mainstream theories, such as creating *Sustainable Value* (benefits for both stakeholders and shareholders), as well as various models for sustainability, including *Doughnut Economic* thinking, the *UN Sustainable Development Goals*, and the *Circular Economy*. We note the contribution of Meadows et al. (1972), who first proposed that there are *“limits to growth”* using a Systems Dynamics model, which influenced the Brundtland Report (1987) that offered an early definition for sustainable development. More recently, there has been a more explicit focus on climate change, noting that all economic and social progress is rooted in not continuing to overshoot planetary boundaries (Weaver et al. 2021 describe this as humanity ‘ultimate’ wicked problem). In this paper, we argue that sustainability, as an emerging paradigm, is often marginalised or overlooked when declaring a worldview. Our contribution is to demonstrate that SSM can be a valuable method for meaningfully engaging communities by triggering conversations on learning (such as to explore new narratives, or justify not to, when engaging with *models for sustainability*). A double-loop learning cycle is proposed to challenge the underlying assumptions of our worldviews and mental models within various stakeholder groups. Particularly, when shifting to higher levels of systems consciousness, by giving (a metaphorical) ‘voice’ to those not present, future generations, and nature itself, which are (or will be) interdependent on communities. Therefore, our research question concerns *how SSM can help support the co-creation of sustainable value through community-based learning?*

## Part 1 Refreshing Soft Systems Thinking Contribution in the Context of Community OR

We begin where Herron et al. (2024) left off, with a series of statements that led to the formulation of refreshed research questions important in developing Community OR practice and theory (listed in Table 1). This came about based on the authors’ experiences and reflections when considering the body of Jackson’s work in systems thinking (with significant contributions in critical systems thinking and Community OR). The concept of “community” is intentionally left open and flexible in Community OR to allow the field to embrace a diversity of collectives of people, stakeholders, their perspectives, and actions. Midgley and Ochoa-Arias (2018, p. 772) suggest that “community” is a label for a variety of people engaged in ongoing learning and debate about community development practices.

**Table 1:** Opening position to refresh ‘Soft’ ideas in SSM in community applications for sustainability

Refreshed research statements for communities (Herron et al. 2024)	
1	Community-based research requires us to go beyond models of ‘interventions’ to models where knowledge is co-produced.
2	Increasing capacity within communities.
3	Emphasis has shifted from ‘giving’ marginalised groups (or absent stakeholders) a voice to creating opportunities for more meaningful and equitable dialogues about what is needed to improve the quality of life within communities.
4	Systems Thinking can be used in different modes—both to guide the design of interactions and/or to help directly shape the concepts individuals and groups articulate.
5	Recognise (as Jackson, 2004, had) that sustainable development may often require the consideration of non-human stakeholders (environment, wildlife/biodiversity, etc.).
6	Community OR requires an ongoing commitment to questioning and refreshing the boundaries of a situation.
7	We still face some ongoing challenges that have existed (albeit in a different form) since the early days of Community OR. However, we also face a range of brand-new challenges and opportunities.
8	We acknowledge that problems (particularly ‘wicked problems’) are not usually solved but often require continued input and energy from all involved.
9	There are many mechanisms by which communities can be engaged.
10	Interventions can often become stuck in the exploration stage, with conversations that appear disconnected from subsequent actions.

The importance of community learning in meaningful dialogue helps facilitate a shared sense of *place* (a geographical location where people and nature are interwoven), *interest* (shared purposes and values), and *identity* (interactions in cultural, social, or political affiliations) (Midgley and Ochoa-Arias 1999). In addition, Covid-19 has vividly demonstrated that *“space”* could be added as a fourth, recognising that people may form interests or identities that transcend physical locations, such as those that might be virtual or even abstract (e.g., a community blog, social media channel, virtual reality). We follow Ulrich (1987), who distinguishes between those *involved* and *affected* by an analysis/action. Those involved can build community learning through *cooperation* and/or *collaboration* and raise awareness of issues/concerns by making boundary judgments that represent those affected by the action. This raises the question of the marginalisation of issues/concerns, considering their pertinence and dependence upon those involved in how a community creates meaning through their interactions. In this way, a community can be seen as a ‘self-producing system,’ where (at least some) meaning is shared, grounded in different interactions through the dialogue of ideas, purposes, interests, and values.

Table 1 presents these original statements, serving as an opening position on how “soft” systems ideas can be enhanced through community actor involvement in analysis/action.

The authors stress Jackson’s (2019, 2020) recognition of the ongoing importance of the concept of ‘emancipation’ and other forms of ethical behaviour (Varela 1999) when collaborating with communities, particularly in considering power dynamics and participation. To do so requires a refresh in the use of systems language in line with that more commonly used by the stakeholders undertaking the analysis and/or action themselves. For instance, Weaver et al. (2025) take a business perspective on viewing purpose, values, and meaning when adopting and incorporating systems thinking principles and practices. Necessary to ensure a *‘meaningful engagement’* with community actors, often marginalised individuals, Community OR should also be concerned with groups or those that represent absent stakeholders, such as the needs of *nature* and *future generations* (further discussed in the following section).

## Part 2 A – The Methodology Underpinning this Paper

The paper follows the methodology adopted by Herron et al. (2024), starting with their revised research statements for advancing Community OR (Table 1), followed by consideration of the context of Checkland and SSM’s contribution when declaring a ‘sustainability’ worldview, drawing on a self-organised process (of both formalised and informal stages, shown in Table 2) that incorporates ideas drawn from a combination of the Delphi method (Dalkey and Helmer 1963) and double-loop learning (Argyris 1976), reflexively so to articulate and combine our ideas in a semi-structured way.

**Table 2:** Research process following a Delphi method and double-loop learning

Phase	Adopted in Herron et al. (2024)	Contextualised for this contribution
*Phase 1* – Initial discussions and agreement on method and data collection/paper protocol		
*Phase 2* – Data collection point 1	Initial points of resonance with Jackson’s work	Review of refreshed research questions that are important in developing community OR practice and theory, as presented in Herron et al. (2024) in the context of: 1) Resonance with the original ‘soft’ ideas of soft systems thinking (*method*) 2) Addressing sustainability challenges in multi-stakeholder action (*application*)
	• Collection and discussion • Identification of initial common themes and an initial framework of ideas • Discussions and identification of candidate vignettes to include concerning the framework of ideas identified	
*Phase 3* – Data collection point 2	Written vignettes of Community OR/systems practice • Collation and discussion	
	• Group members’ reading of Jackson’s recent writing for further discussion • Contributions and iterations of the emerging paper text	‘Group members’ reading of recent applications of soft systems thinking in community applications for further discussion • Contributions and iterations of the emerging paper text
*Phase 4* – Data collection point 3	Identification of statements of importance and new directions • Collation and discussion	
	• Reading/sharing Jackson’s recent writings – Further discussion of resonance	• Reading/sharing of more recent developments in the use of ‘soft’ ideas emerging from declaring worldviews for complex real-world organisational sustainability challenges
	• Contributions and iterations of the emerging text	

*Phase 5* – Editing and extending (and curtailing!) the emerging paper

Data collection began with recognising ourselves as suitable individuals to consult. Each of the papers’ authors has contributed to the ‘Community OR Stream’ at the UK Operational Research Society conferences and has a track record of applying SSM in practice. Our expertise lies in co-creating sustainable value through community-based learning using soft systems ideas and SSM.

The process protocols followed (see Table 2) were intended to be fluid (often non-linear, as Table 2 may appear) and iterative. Information and ideas flowed between us, both formally and informally, as we attempted to reflect on and articulate how reflecting upon a sustainability worldview in SSM applications has helped bring about more resources in communities. We found it critical to see the emerging paper as a living document (available for editing and developing ideas) as a collective common. However, as was found in Herron et al. (2024), the distinct rounds of data collection (on clearly defined themes) helped us to keep focused, enabled distinct individual contributions, avoided excessive divergence, and encouraged a convergence of ideas between us without being too limited. We take the perspective that the ideas of Soft Systems and Community OR, which have contributed to emancipation, now resonate and are critical to tackling sustainability challenges.

## Part 2B: the Initial Framework of Ideas

An initial framework of ideas was created following the author’s reflections on Herron et al. (2024), refreshed research questions for developing Community OR in the context of this paper: (1) community learning using ‘soft’ ideas of soft systems thinking with SSM (*method*) and (2) addressing multi-stakeholder sustainability challenges (*application*). The three key ideas to be explained in this section include:

- *Importance of meaningful engagement of communities in an OR/systems intervention in declaring a worldview* (Section 4.1).
- *Sustainability as a worldview is often marginalised or ignored in problem structuring*,* yet pertinent to the legitimacy of any System of interest* (Section 4.2).
- *SSM is a valuable tool to support community engagement*,* participation and learning to find common ground before taking meaningful action* (Section 4.3).

### Engaging Communities in Declaring Worldviews

SSM has long been established as a method that incorporates a continuous learning cycle (Checkland and Poulter 2006). Particularly useful for addressing complex, messy, and ill-defined problem situations that concern multiple stakeholder perspectives (Checkland 1999; Petropoulos et al. 2024). These problems are often described as “wicked” (Rittel and Webber 1973). They are challenging or even impossible to solve due to the competing purposes and values of different stakeholders, often in conflict, which makes it difficult to find common ground for taking meaningful action (Ackoff 1974; Midgley 2000). Therefore, the structured approach offered by SSM is seen as valuable in learning about the problem situation in its real-world context, framing worldviews from multiple stakeholder perspectives, building mental models, and debating desirable and feasible change (Checkland and Poulter 2006).

It is the *worldview* (or, as Checkland more specifically expressed, the *‘Weltanschauung*,*’* with its more profound meaning in the German language) that gives meaning to *‘*what the System does*’* (its ‘transformation process’). Weaver et al. (2025) suggest this is more akin to contextualising a system “why” yet recognising that *“Systems do not have purposes*,* people do”.* The exploring stage in SSM helps to surface and declare a worldview that the models of relevant purposeful activity systems (the conceptual models) are based on. New learning is unfolded and bounded by who participates in the analysis and how they represent themselves, and importantly, by the stakeholders deemed relevant, their issues/concerns, and their values. SSM, as a learning cycle, is powerful; however, the scope and quality of the action to improve the situation (the intervention itself) are highly dependent on who participates and what they consider relevant. This influences the formation of a declared worldview and the subsequent models generated based upon it. The authors suggest that this is critical in analysis and can become problematic when reflecting on often marginalised worldviews such as that presented by a sustainability paradigm.

### Sustainability as a Worldview Is Often Marginalised or Ignored

The sustainability paradigm has gained widespread recognition for its importance in both theory and practice, yet it is often marginalised, overlooked, or ignored. This is at odds with the prevalent and dominant neoliberal paradigm, which emphasises the role of free markets, limited state intervention, and individual responsibility as drivers of economic growth and social progress (Oxford University Press, n.d.). This neoliberal paradigm has given rise to trends in the 1980 s, 1990 s, and beyond, including market liberation, deregulation, privatisation of previously state-owned enterprises, competition, globalisation, and growth at all costs in predominantly Western populations (Metcalf 2017). Although associated with social progress, its critics point to rises in health and social inequalities, disinvestment in public services, and erosion of employment rights and representation, among other concerns (Bell and Green 2016). Additionally, the natural resources required by industry for inputs are degraded, resulting in considerable biodiversity loss (Ceglar et al. 2024), drawing new meaning to Hardin, (1968) illustration of the *‘Tragedy of the Commons’* as a phenomenon. As Meadows et al. (1972) aptly identified over 50 years ago, the *“limits to growth”* will lead to inevitable collapse when reached. It is in the interest of humanity to reflect on these concerns and develop solutions that aim to decarbonise (D’Arcangelo et al. 2022), while also protecting nature and fostering more regenerative and redistributive economies (Raworth 2017).

A shift towards a sustainability paradigm necessitates a critical and reflective practice that challenges the underlying assumptions of policy, theory, and practice by questioning deeply rooted economic and industrial norms. Models for sustainability include *The Doughnut* (Raworth 2017), the *UN SDGs* (United Nations 2015), and the *Circular Economy* (Ellen MacArthur, 2013). The *ethical imperative* for sustainability is aptly captured by Raworth (2017) in “Doughnut Economics”, now part of mainstream management thought, which aims to *“meet the needs of all people within the constraints of a living planet”.* The Doughnut (2017) shows how this can be considered based upon two critical boundaries: (1) not to *overshoot our ecological ceiling* and (2) not to *shortfall our social foundation* for human wellbeing. Weaver et al. (2025) place this ethical imperative within a systems context, recognising that *all human activity systems are interdependent on the abundance of natural resources and nature’s ability to regenerate*. This challenges the legitimacy of all human and living systems’ ability to thrive in perpetuity. This builds upon the seventeen United Nations Sustainable Development Goals (UN SDGs), which are to be achieved by 2030. Following Ackoff (1974; 1993), the UN SDGs should be viewed as ‘ideals’ that demonstrate the ‘wickedness’ of sustainability as a challenge, for instance, achieving one goal at the expense of another, highlighting the deep interconnectivity between goals and actions. Recent reports by the United Nations (UN SDG Report, 2024) show that only around 17% of these goals are on track, with nearly half showing minimal or moderate progress, and many having stalled or regressed. While the concept of the circular economy is also a model for sustainability, it is deeply rooted in systems thinking and approaches (Pokorna et al. 2024), offering three key principles for its application. These include *eliminating waste and pollution*,* circulating products and materials*, and* regenerating* *nature* (Ellen MacArthur Foundation 2024). The *‘eliminate’* principle has long been practised as part of other popular management philosophies such as lean (Ohno 1988) and TQM (Deming 1986), with an emphasis on reducing costs and increasing customer satisfaction. This thinking follows traditional economic logic, whereas the other two principles necessitate a different mindset and a shift towards an ecosystem-level awareness.

These three models for sustainability offer insight into the ethical imperative, as well as the challenges and difficulties in their application. The authors recognise that declaring sustainability as part of a worldview is challenging, and its continued marginalisation, overlooking, or lack of meaning by different stakeholders and communities needs attention in OR/systems research and practice. We highlight that Checkland (1981) stressed that the root definition informs a conceptual model of a purposeful activity system, shaped by its worldview. An SSM analysis is strengthened by the inclusion of relevant participants who represent the perspectives that capture its real-world context. Making judgments on ‘relevance’ (and by whom) is a critical consideration in SSM that will benefit from gaining insights by exploring appropriate models for sustainability as part of community learning to address this challenge. OR/systems research and practice will play a considerable role in addressing our sustainability challenge. With its interdisciplinary nature, long-standing history of addressing grand challenges, and systemic orientation (Lane 2010; Stowell 2018). This will not only strengthen the understanding of the real-world context but is likely to lead to more *meaningful* action. On the other hand, if not, it is also necessary to understand the consequences of not acting, whether it is too little or too late.

### Supporting Community Learning to Find Common Ground

The previous two sections first stress SSM as a learning cycle and, secondly, emphasise the dependency of this learning on the unfolding and declaration of a worldview. This paper’s contribution lies in the field of Community OR, with an increasing interest in community engagement for addressing sustainability. For sustainability to shape a worldview and be relevant in communities, any narratives that arise from a critical engagement with sustainability insights must be made applicable and internalised within the context of the communities themselves. From our collective experience in teaching sustainability models, the authors have found that learners often find such insights challenging, without engaging in critical reflection on how they perceive their framing and mental models for sustainability as an ethical imperative, challenge, and application. SSM can play a role here by triggering conversations and collective understanding as part of the discovery of meaning taken from learning about sustainability and challenging underlying assumptions. Using Searle’s (2006, p. 12) term, these can be described as ‘status functions’ (the glue that holds society together, as they create deontic powers, powers that work by creating desire-independent reasons for action). In systems language, we challenge our mental models and worldviews. This will help recognise the different perspectives held by communities and how they clash with stakeholders affected by the action, towards the legitimisation of a worldview.

A difficulty in adopting a sustainability paradigm requires a shift in systems consciousness, recognising the needs of nature and intergenerational interests in building sustainable futures (Weaver et al. 2025). This representation is problematic, as it requires those involved in an analysis to offer a metaphoric voice for nature and future generations who are not present. Weaver et al. (2025) suggests an upfront and continual boundary critique for sustainability, by reflecting the purposes and values that represent four levels of systems consciousness with: *oneself* (the “ego”); *others* (communities, co-constructed through dialogue, participation, and shared inquiry, Midgley et al. 2017); *nature* (an ecosystem awareness) and *intergenerational* (existing and future generations). By questioning the underlying assumptions of the declared worldviews and considering distinct levels of system consciousness, the authors suggest that new meaning can emerge for relevant stakeholders, their pertinent issues/concerns, and their values. Nevertheless, the shift from an *ego* (an anthropocentric view) to *ecosystem awareness* and a futuristic outlook will help question a system’s legitimacy (as part of a system’s viability to sustain itself). Additionally, recognising the interdependence between human and living systems and emphasising the thrivability of Systems (to self-produce and be regenerative) may, to some extent, address the ethical imperative. Considering that Systems’ *legitimacy* and *interdependence* have been identified by Weaver et al. (2025) as critical principles for applying systems thinking to sustainability, we now suggest that SSM can be enhanced by creating new narratives with participants as part of community learning, using appropriate models for sustainability.

## Part 3 A: The Vignettes – The Initial Framework of Ideas

The five vignettes (one offered by each author) demonstrate a variety of approaches and application areas. Nevertheless, all use SSM as their core method and seek to embrace the range of soft systems ideas within the theme of building community learning. Like Herron et al. (2024), we draw on Vilalta-Perdomo and Salinas-Navarro’s (2023) *‘Communities*,* Roles*,* Methods’* distinctions for discussing Community OR.

### Communities

The supported communities were diverse in terms of both their local (two city-level, one regional) and one at national level, including one in a developing country and another in a rural context. Three of the cases took a more diverse set of stakeholders (a combination of residents, businesses, government officials and third sector organisations). The international and rural example represents a more traditional Community OR application concerning a marginalised neighbourhood. Three cases explicitly concern absent and/or non-human stakeholders (i.e., nature and future generations) and the interconnectedness of an organisation within its socio-environmental system. Two cases explore broader stakeholder-oriented concepts that are mainstream in the sustainability field, namely *Sustainable Value Creation* (Hart and Milstein 2003) and the *Circular Economy* (Ellen MacArthur Foundation 2013).

### Roles

The academic partners were acting in a way to support learning; either the learning of community members directly (as part of a *civic* engagement of a university) or indirectly through student learning (involving academics as part of a *teaching* role in a university) or a supply chain (applying *research* expertise to address a real-world challenge, foster innovation and impact). Providing the necessary structure to support co-creating meaningful action through sharing understanding and meaning, building commitment and trust, and a collective consciousness.

### Methods

The cases were selected as they employed SSM as the core approach within community applications addressing a sustainability challenge. The teaching case illustrated how participants perceived SSM in comparison to the Viable System Model in the context of military training. In contrast, the Scotland case adopted a multi-methodology approach that included an upfront boundary critique, using the *‘Systemic Sustainability’* framework (presented in Weaver et al. 2025), which incorporates ideas noted in Section 4.3.

Vignette 1: Evaluating mobile education in a rural community.

**Table:** 

In the early 2000s, researchers at CORU were invited to evaluate the work of a mobile education/youth work bus that visited educational sites across a rural county. These included mainstream secondary schools and sites where young people were educated outside of mainstream settings. At one of these external settings, we used a form of SSM for data collection. We encouraged several participants to draw Rich Pictures, and we used CATWOE as part of our process to gain a deeper understanding of the situation.
One experience stood out during this process. We were sitting on the bus with two teenage boys, drawing a Rich Picture (we called it a Rich Cartoon, as we were not expecting a full articulation of stakeholders but instead wanted to explore the aspects of importance to the young people we were talking to). We asked them to draw the bus in the centre and then draw the things and people they associated with it around it. They introduced the people involved (including the youth worker, on-site tutors, and the bus driver) and explained their roles. They also drew many related inanimate objects (e.g., the drug-awareness software and the computers on the bus, as well as the car workshop and tools on the site, which were only indirectly connected). They were enjoying themselves and laughing together as they drew all the characters, but the most remarkable point was when they drew a horse. At this point, they looked up at us for the first time and said that this was "the Killer Shetland Pony" and would we like them to show it to us. We left drawing the Rich Picture at that point and were then taken on a tour of the site, which included the ponies and the car workshop.

Comment: *The drawings made by all the different stakeholders as Rich Pictures were all valuable for our evaluation; the one made by the two teenagers was particularly valuable for explaining to us how at least some of the users of the service perceived the relationships between people and inanimate objects involved (including the bus itself). It helped us to understand the situation in a way that we would not have been able to with a more traditional interview method. The main advantage was that it employed a different form of communication (non-verbal and graphical) and helped break down the evident communication barriers*,* as the young people talked in a more relaxed manner about what they were drawing and then felt confident to speak and show us around their site. It also allowed them*,* in an unforced*,* natural way*,* to introduce other non-human actors into consideration who would not otherwise have had a voice yet were particularly important in their understanding of why the setting worked well for them. Once the young people had made this point so clearly*,* we could also see it in the Rich Pictures that other stakeholders completed. We would argue that this is a feature of using SSM that we found particularly valuable.*

Vignette 2: The challenges of Pueblo de Santa Fe and the work of Centro MAPFRE-Universidad Panamericana:

**Table:** 

Pueblo de Santa Fe is a marginalised neighbourhood in western Mexico City, facing serious economic, social, and environmental problems. Located near the wealthy business district of Santa Fe, the community struggles with poverty, violence, environmental degradation, and a lack of basic services. Most residents have informal jobs with low pay or work endless hours to cover their basic needs, while others face unemployment. Children often drop out of school due to economic pressures or the influence of local gangs. Access to healthcare is limited, and public infrastructures, such as roads, water, and electricity, are often unreliable and of inadequate quality. Additionally, poor air quality and contaminated water sources exacerbate the health issues faced by residents.
Amid these difficulties, the Centro MAPFRE-Universidad Panamericana serves as a vital community hub, providing essential support to the community through basic medical services, free meals, educational support, and cultural workshops. However, the question now is: *How can the centre take the next step? How can they build trust, prioritise the most urgent needs, and get more people working together?*
**Using SSM in the Community**
Staff from the Centro MAPFRE-Universidad Panamericana gathered with researchers to jointly enhance community engagement and social impact in Pueblo Santa Fe. Using SSM, they expressed the problem situation (using a rich picture), discussed concerns (such as unsafe streets, children dropping out of school, and a lack of medical clinics) (to identify potential interventions), and defined relevant system definitions (using X-Y-Z root definitions and the CATWOE tool). "We want to help, but we don’t have a clear picture of the situation and know where to start," admitted one volunteer.
Participants collaboratively explored the current situation of Pueblo Santa Fe. They shared their views and described relevant stakeholders, their concerns, community roles, values, and norms, as well as conflicts and agreements. Later, participants drew a rich picture as a visual map of the issues, relationships, and stakeholders. Accordingly, based on a worldview of ‘community transformation through collective engagement, commitment and participation,’ four tentative interventions were envisaged based on the current limitations and capabilities: • A system to develop lifelong skills through community workshops and training to enhance employability. A system to grow social skills for peaceful and safe civic coexistence, through on-site development programmes, classes, and workshops, to further reproduce positive social behaviours. • A system to provide legal and psychological support through community counselling and advice services to facilitate violence prevention and justice access. • A system to identify healthcare community requirements through resource planning and community consultation to increase and enhance the current medical service offer.The collective worldview here stressed that social change is possible through collective understanding, action, and participation, enabling social awareness and learning.

Comment: *The researchers engaged with the community centre*,* aiming to create a mechanism for interaction that would produce social impact and enable experiences of both internal and external learning. A key takeaway is evident in the community centre staff’s ability to articulate their distinctions and meanings into a collective understanding and knowledge. Nevertheless*,* people recognise that*,* beyond their collective desirability*,* internal structural limitations and external constraints exist that impinge on their efforts. Therefore*,* an SSM implementation does not only depend on people’s participation and engagement but also on the contextual circumstances (and constraints) in which they are immersed.*

Vignette 3: Co-creating shared spaces for cross-sector collaboration.

**Table:** 

Between 2015 and 2017, a Knowledge Transfer Partnership was established between a Scottish fund manager and Edinburgh Napier University. The project, employing a Community OR approach using SSM, was conducted to investigate how third-sector fund managers can more effectively engage with the private sector to address existing and emerging community challenges. Over 240 participants from Scottish businesses, the third sector, fund managers, umbrella bodies, local and national government, their agencies, and public sector bodies participated in problem structuring. Rich Picture analysis showed a perceived lack of connectivity and alignment of objectives between and within public, for-profit, and third sectors in Scotland.
Following this purposeful activity, models were constructed based on declared worldviews. The *‘Connect model’* was proposed to help bring more resources into communities, particularly from the private sector. The Connect model asserts that there is both a need to invest in building social capital between stakeholders (a fundamental early intervention) and to establish a ‘shared space’ where businesses, communities and citizens can come together to collaborate on initiatives and actions. The shared space concept recognises that, as the for-profit sector controls most resources, they need to be part of any solutions. Nevertheless, it also incorporates the view that, for businesses to fulfil their role as responsible actors in society, they need to empower communities to identify issues and be part of co-creating the solutions. The ‘Responsible Business Forum’ was launched in 2016 in collaboration with the Scottish government and its development agencies, aimed at bringing together businesses and communities in an ‘open system’ that addresses the entire problem area. The University and fund manager were seen as conduits who could act by unlocking and facilitating shared space based on a match of shared aspirations and values.
Overall, the connect model worldview was influenced by the opportunity to release resources into communities from the private sector, as they control most resources in most economies. However, this required an approach that offered an alternative to the neoliberal business paradigm (the dominant worldview in business and society), one that must involve the inclusion of people and communities, in part represented by third-sector organisations. This led to the refinement of the Connect model worldview and how this gives meaning to the transformation process with new learning. The five transformational processes are considered from the perspective of 1) the fund manager itself; 2) third sector organisations; 3) Scottish Government and agencies; 4) for-profits; and 5) concerned citizens.
Later, the Connect model was scaled to adopt a place-based approach at the city level (Edinburgh) when the researchers, along with other city stakeholders, successfully applied to become Europe’s first designated CAN-B city. CAN-B exists as a global movement designed to mobilise hundreds of thousands of people (citizens and organisations) to collaborate in pursuit of the UN Sustainable Development Goals (UNSDGs). Edinburgh CAN-B is now a registered charity supporting a range of community-led issues and challenges with partner businesses that provide resources and capability in pursuit of creating sustainable value (to benefit all stakeholders deemed relevant in the analysis).

Comment: *The researchers assisted a Scottish grant-maker in exploring the prevalent neoliberal paradigm and seeking new ways to release more resources from for-profits into communities through the creation of joint business and community value (seen as finding common ground for WIN-WINs by both parties). This required the development of new capabilities to function as a conduit*,* unlocking and creating shared spaces where co-creation could occur. The client rebranded from a grant-maker to an intelligent fund manager*,* shifting its focus from collecting and allocating funds based on funder criteria to one that supports community learning in co-creation processes. This required building social capital through events and relationship-building*,* appreciating the issues and challenges faced by business and third-sector organisations (that represent community interests)*,* and facilitating partnership working towards shared goals and values*,* taking account of various stakeholder worldviews.*

Vignette 4: Challenge-based learning and SSM in an executive MBA module.

**Table:** 

Following the strategic aims of a British HEI that aims at developing a closer relationship with the communities that it serves, a group of academics designed and delivered an Executive MBA module, following the principles of Challenge-Based Learning (CBL) teaching and learning (T&L) approach. CBL is an active learning approach where students’ teams are challenged to design and manage effective and efficient operations, systems, and processes within a real-world organisation. CBL comprises three phases: (1) engage, (2) investigate, and (3) act, where students document their experience and reflect on their learning.
For every edition of the module, challenges change and involve a variety of complex situations, such as dealing with Bangladeshi restaurants multiple constraints, defining the operational conditions of an airborne early warning and control system (AWACS), building a collaborative organisational structure where a community of micro-businesses might evolve into a resilient and sustainable food supply chain, or developing a novel commercial business model that enables a housing association to diversify its funding sources.
The module equips students with two systemic approaches to build conversations that help them to reflect on their proposals in terms of: -*How can we organise conversations in such a way that collectives and their members will improve simultaneously?* *-What value do these conversations provide?*
SSM was the first approach used to help students’ teams make their views explicit and accommodate them in proposing organisations with a coordinated collective action for improving their operations, systems, and processes. The second approach provided was the Viable System Model (VSM). VSM is used to support students’ teams in designing more viable organisational structures that can effectively respond to internal and external complexities.
The experiences collected from running this module suggest that both approaches (SSM and VSM) help support the meaningful co-creation of sustainable value. They are also effective in providing stakeholders with practical actions to address sustainability challenges. Finally, they support individual and community learning. However, SSM and VSM seem to be better understood and more effectively embedded in individuals’ discussions, depending on their professional background. For instance, students who work in a vague environment, where no specific definition of the problems is provided and clear instructions on how to act are not given, seem to prefer using SSM. Conversely, students in more rigid structures (e.g., military branches or manufacturing) tend to prefer VSM.
Our hypothesis, based on what we have observed and the literature, is that: -SSM appears to be more effective in studying human actions, where it is sometimes necessary to acknowledge conflicting purposes that participants may have explicitly, and a consensus needs to be established for collective success. -VSM is more effective when the focus is on designing structures that enhance an organisation’s capacity to adapt to internal and external risks, while strengthening its ability to remain aligned with its environment.

Comment: *SSM is more effectively received by individuals with diverse agendas who must work together to achieve coordinated actions. In this sense*,* it can be argued that SSM is suitable for supporting collective learning in ambiguous milieus. Indeed*,* this needs to be tested so that it will be developed in future stages of our collective research programme.*

Vignette 5: Supporting community learning towards circular supply chains.

**Table:** 

A leading construction company approached the research team with a clear objective: to strengthen the sustainability maturity of its supply chain. The company had already introduced an internal classification framework, bronze, silver, and gold tiers, and now aimed to support its Silver-tier suppliers in progressing toward the gold standard.
We initiated our collaboration with a keen interest in understanding how these suppliers perceived and addressed the challenges related to sustainability and circularity. To explore this, we proposed a workshop based on SSM, which we believed offered an effective way to navigate the complex dynamics of the sustainable supply chain. The workshop revolved around a key question: *What challenges are you facing to be more sustainable to help the planet restore and shift to a regenerative future?* Participants were asked to express their views using a rich picture, a technique designed to capture the complexity and ambiguity of the situation. Even though participants had been provided with a working definition of sustainability and circularity, many expressed uncertainty and confusion. Their illustrations revealed a lack of clarity, and several admitted they were unsure what was expected of them. Some seemed hesitant or even uneasy, requesting further explanation of the corporate purchasing team's goals.
This initial uncertainty highlighted the relevance of the SSM approach. Drawing on Fonseca et al. (2023) framework, we began by “making sense of the mess.” The session became a space for shared learning, structured around seventeen guiding questions linked to environmental themes and selected Sustainable Development Goals (SDGs). Topics such as Scope 2 emissions served as conversation starters, encouraging deeper reflection and discussion.
Throughout the workshop, we returned to the rich pictures, not to revise them, but to reinterpret them, considering new insights. This repeated dialogue allowed participants to build on their understanding. That said, we later recognised that the workshop remained within the space of single-loop learning, focusing on improvements without critically examining the underlying assumptions. Double-loop learning, which involves rethinking core beliefs and values, remained out of reach in this setting.
One concrete outcome emerged from a discussion centred on plasterboard. This material became a crucial point for exploring circular design strategies. While reducing waste in this area proved straightforward, tackling broader issues, such as thinking beyond “simple” recycling or the principle of regenerating natural systems, posed more significant challenges. These reflections highlighted the need for an ongoing, adaptive learning process.
In reflection, the workshop marked an important starting point. It created space for open dialogue and raised doubts that are often overlooked. While it did not deliver a profound transformation, it set the stage for it. The experience demonstrated that real progress toward circularity depends not just on technical solutions, but on building a culture of investigation, openness, and collective learning.

Comment: *This vignette illustrates the value of SSM in exploring marginalised viewpoints and assessing levels of initial understanding. The rich picture was further developed by introducing new narratives through the invitation of relevant speakers who represent different stakeholder views*,* and by exploring circular economy principles and the Butterfly diagram to surface new learning and meaning. It was evident in the room that the supply chain partners struggled to align with the corporate purchasing team’s intentions*,* which created tension alongside a lack of understanding of the underlying meaning behind the concept of a circular economy among the actual suppliers themselves. Following a series of dialogues with invited speakers*,* including some academic insights*,* a baseline was established*,* and the corporate purchasing team presented a range of opportunities for further engagement. This led to some common ground being found and an agreement on next steps. The process demonstrated how SSM can support mindset shifts by opening doors for reflections and dialogues on making boundary distinctions and learning.*

## Part 4: Discussion on SSM in Community OR Applications for Sustainability

An SSM study begins by exploring and understanding the problem situation from multiple perspectives before taking action. In each of the vignette examples, a “rich picture” was used to make sense of the complex and messy situation, considering the different perspectives of stakeholders, their values, and concerns. The authors emphasise the importance of exploring the meaning behind pictorial representation and its interconnections. The analyst, along with those involved in the analysis, was able to explore ‘boundary’ distinctions by observing the issues and concerns that arose and identifying who was included in the situation. We recognise that the application of rich pictures cannot claim to offer a comprehensive boundary critique. Examining who might be affected and issues/concerns that are also excluded in the analysis. This would require additional systems-based methods and consideration of alternative systemic perspectives, as stressed by Jackson (2021, 2024), as part of a multimethodological approach to gain a richer understanding. However, in each of the vignette cases, the analyst was able to begin supporting community learning by understanding the underlying assumptions of the worldviews and mental models of those involved in the analysis. Three areas are identified that could be considered as part of exploring a problem situation to reflect on sustainability: (1) *Explore worldviews & mental models*; (2) *Triggering conversations on learning*; (3) *Bridge the value(s)-action gap* (double-loop learning); and (4) *find common ground*. The authors suggest that action is more ‘meaningful’ when viewpoints and values are shared, articulated, and can be demonstrated; common ground for action can be found.

### Explore Worldviews and Mental Models

Before an intervention, it is necessary to explore the problem situation in its perceived real-world context. This *‘finding out’* is the first phase (of the four, as depicted in the revised SSM framework, Checkland and Poulter 2006), which historically has used ‘rich picturing’ as the primary method to express the problem situation (See Checkland 1981; Checkland and Scholes 1999). The rich picture draws in the perspectives of the participants undertaking the exercise, as is the case in each of these papers’ vignettes. This is preceded by *‘model generation’* (Phase 2), which involves generating models of relevant, purposeful activity systems, each based on a declared worldview. These worldviews are surfaced in the *‘finding out’* phase, and it is this *declared worldview that gives meaning to what the system does* (the system’s transformation process).

The modelling stage is highly dependent upon surfacing a worldview that can be agreed upon. Phase 3 of SSM concerns *‘comparison & accommodation’* by questioning the problem situation (from phase 1) using the models. Likewise, this ‘real-world’ understanding is taken as given from the perspectives surfaced by the participants in phase 1. Although SSM is regarded as a continuous learning cycle, it provides a single loop of learning focused on encouraging debate to seek accommodations about desired and feasible change.

### Triggering Conversations on Learning

SSM was not intended to explicitly support those involved in an analysis to make boundary distinctions, other than to bound the problem situation from their perspective. This influences the declaration of worldviews, the mental models they derive from, or the accommodations debated before acting. This has given rise to alternative approaches being developed, grounded in the development of emancipatory systems thinking, and at the same time, the prominence of Community OR as a distinct OR/systems subject area. This includes Midgley’s (2000) *Systemic Intervention* framework, which incorporates an upfront boundary critique as part of its analysis, and Weaver et al.‘s (2025) Systemic Sustainability framework, which also incorporates boundary critique ideas from Ulrich’s Critical Systems Heuristics, among others. To some extent, Jackson’s (2024) EPIC framework addresses the need to take alternative systemic perspectives (i.e., mechanical, interrelationships, organismic, purposeful, and societal/environmental) to help and bound the problematic situation in its actual real-world context. Jackson (2021) then draws on a host of systems-based approaches, including problem structuring methods, which can help incorporate such perspectives. Taking an ecosystem and intergenerational perspective aligns neatly with the environmental/societal systemic perspective, providing new meaning to a system’s *purposefulness*.

SSM has a particular strength in taking an *‘interrelationship’* systemic perspective and supports a continuous learning cycle. The problem situation, worldviews, mental models, and actions taken using an SSM process are grounded in the context of how participants identify issues/concerns, values, and their connections. In each of the authors’ vignettes, SSM was used primarily to explore the issues and values of communities that are often marginalised and/or overlooked. Again, this learning surfaced among those involved in the analysis, with some reflection (when participants raised concerns) on *those affected*. In two of the cases, environmental concerns were present, but the focus was on resource availability and consumption, reflecting a more anthropocentric view. At a surface level, this corresponds with taking a social/environmental perspective but did not adequately challenge the issues/concerns that might be pertinent to organisational viability (e.g., carbon footprint, biodiversity loss), and a community social foundation (e.g., health, education), as such issues have yet to be seen as relevant. We contend that a sustainability worldview cannot be forced upon participants. However, SSM allows for the triggering of conversations that facilitate new learning, which can be supported by exploring different narratives with appropriate models for sustainability.

### Bridging the Value(s)-action Gap

OR/systems has a long tradition of bringing about action. The authors here wish to make a distinction that such ‘interventions’ that incorporate a sustainability worldview should be based on ‘co-creation,’ requiring processes of learning in communities. The vignettes demonstrate that SSM has been a successful approach for incorporating multiple perspectives, exploring meaning, and building narratives that facilitate meaningful action. However, as noted in the previous section, this depends on who participates in the analysis, the issues they include, and the values they share. SSM may not necessarily surface all the pertinent issues between the primary and secondary boundary and the system’s wider environment. This raises the concern of highlighting the issues and values that ought to be relevant to exploring the problem situation, which may not have surfaced but influence the *legitimacy* of the system in question.

We propose that a *value(s)-action gap* exists when declaring a sustainability worldview (or indeed any other) like those explored in strategy making (e.g., Tsoukas 2018; Williams and Preston 2018), marketing (e.g., Kumar and Reinartz 2016; Chamberlin and Boks 2018), and environmental geography (Ribeiro et al., 2025), among others. The value(s)-action gap (or intention-behaviour/action gap) examines the discrepancy between an organisation’s stated values and actions, specifically the difference between what people say they value and what they do (Blake 1999). This phenomenon has yet to be explored in the context of sustainability and net-zero applications. For example, awareness and concern for environmental issues are growing, but have not yet led to a similar meaningful engagement in taking responsibility and action. Likewise, purpose-led organisations (those with a mission and value statement that underpins their social/environmental impacts) have a strong “digital footprint” that demonstrates progress in sustainability and net-zero measures, leading to meaningful action aligned with their values —a positive values-action correlation. Organisations lacking purpose and values in sustainability and net-zero (e.g., environmental sustainability, ecological stewardship, resourcefulness, regeneration) tend to have poorer outcomes (Fonseca et al. 2023).

Weaver et al. (2025) noted that an emphasis on the need to put *values into meaningful action* requires balancing purpose (both value and values judgments) with an organisation’s impact. An organisation’s purpose comprises both value and value judgments, which are the value created for those involved in taking action and the alignment of issues that bring people together, as well as the values that make it meaningful. This, to some extent, aligns with Checkland’s (1999) concept of the transformation process (the ‘what’) and worldview that gives meaning to this transformation (more akin to our understanding of the ‘why’ an organisation/collective exists), yet uses established business terminology. Impact is felt in communities, which Weaver et al. (2025) note are those affected by the action in *places* and at the right *pace*, going to the heart of WHY organisations do WHAT they do (or not). By using more accepted business terminology and understanding, emphasis on purpose-making (shared issues of concern, balancing value with value judgments) and interventions grounded in processes for co-creation will lead to better intention-setting linked to meaningful action (value and impact created for the organisation and its stakeholders).

The *value(s)-action gap* can also be linked to the systemic ideas of management cybernetics (Espejo et al., 1999; Salinas-Navarro 2010). To achieve effective coordination and action, people need to establish the requisite structures that support their individual and collective needs. People continuously and recursively ground their meanings in their moment-to-moment interactions, forming relationships and (re-)crafting their ideas. Regarding sustainability ideas in communities, this proposition suggests that conversations must be triggered to incorporate sustainability-related notions into people’s everyday language and meanings. However, to make things happen, communities must also build the necessary structures of relations and resources to enable the production of their meaning. Therefore, sustainability narratives must achieve collective understanding and shared meanings while developing practicality and operational support. Failing to do this might produce values without action or action without values. This idea needs further investigation within the Community OR.

### Find Common Ground

The *value(s)-action gap* can be described as a phenomenon that needs to be bridged in problem structuring when declaring the worldviews that give meaning to our mental models. Such a view supports double-loop learning by challenging the underlying assumptions of these worldviews and mental models, thereby contributing to community learning. This supports the final phase of SSM by making accommodations that enable actions to improve the situation. However, at this deeper level, meaningful dialogue around alternative narratives, agency, and voice (including the representation of absent stakeholders) is supported to explore the shared understanding and meaning of the issues and values that shape a declared worldview. This shared understanding is central to the co-creation of new knowledge, as well as building trust and reciprocity among the community (Pearce et al. 2022). This was evident across the cases but required setting good reflective questions to initiate a rich picturing workshop that explored the problem situation and involved diverse participants who should represent marginalised stakeholders, issues/concerns, and their values. This depended on the participants offering representations if deemed relevant. Often, this is a primary reason interventions fail – they lack the co-creation of action rooted in a thorough exploration of the real-world problem, as perceived from various stakeholders, and taking into account boundary judgements that have yet to surface.

## Proposed Model To Support Community Learning in SSM

Exploring the *value(s)-action gap* and how it can be bridged as a phenomenon through continual reflection on alternative narratives can help develop common ground as a precursor to collaboration or cooperation, whether it involves places, spaces, interests, and/or identities. As previously noted, soft system ideas have influenced the need for exploring and structuring problem situations. Nevertheless, does not explicitly address making boundary distinctions that distinguish between the primary and secondary boundary issues and values. Reflecting upon the value(s)-action gap as a phenomenon for exploring sustainability to shape the worldview may offer a useful upfront boundary distinction in understanding the complexity of a messy problem situation.

**Fig. 1:**
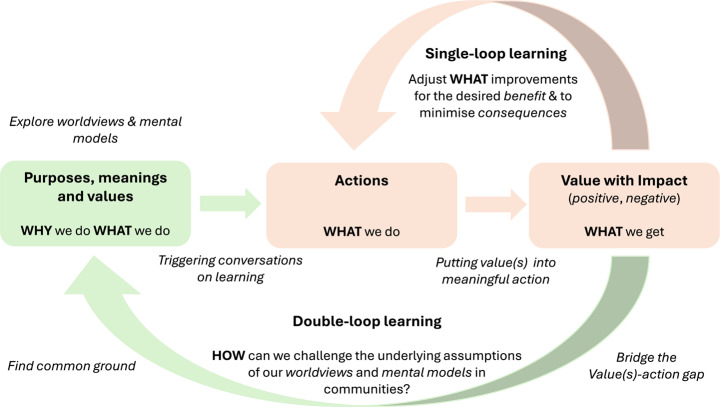
Supporting double-loop learning in Community OR applications using SSM

Organisational and community learning has been a well-researched topic, with seminal works by Argyris and Schön (1978) leading to the concepts of *single-* and *double-loop learning* in the late 1970s. In single-looplearning, organisations and/or communities may modify their actions based on the difference between desired and actual outcomes. For example, an organisation may evaluate its food waste situation and make improvements by adapting, reinforcing, and/or balancing its behaviour, and taking corrective action. In single-loop learning, the most pressing issues are addressed by removing the highlighted symptoms, but not necessarily the root causes. Sustainability, by its nature, inherently contains numerous interconnected wicked problems that are difficult or even impossible to resolve. This requires double-loop learning, by examining the underlying assumptions and meanings that address why an organisation, or community, does what it does (its intentions, as manifested in its purposes and values), and reflecting on the boundary distinctions described by the value(s)-action gap phenomenon. This single and double loop learning cycle is represented in Fig. 1, highlighting the potential dialogues that could support community learning by exploring worldviews and mental models through reflection on critical boundary distinctions for sustainability, as well as understanding reinforcing and balancing behaviours.

The distinction between single and double loop learning, following SSM (or even the Jackson ‘explore’ stage in EPIC), may help lead to more meaningful interventions that have emerged through an ongoing process of co-creation and learning. Sustainability as a worldview is most concerned with double-loop learning, achieved through deliberate reflection on pertinent issues within, between, and across primary and secondary boundaries, as well as the broader systems environment. Building capacity for double-loop learning in SSM is a central contribution of this paper, as it highlights the need to incorporate often-marginalised or overlooked issues and values and their necessary supporting structures that are deeply interconnected to ensure the legitimacy of a given system.

## Conclusion and Future Research Directions

Soft System Methodology (SSM) is a valuable method to *support the co-creation of sustainable value through community-based learning.* This paper suggests SSM can be enhanced by adopting a double-loop learning cycle to challenge the underlying assumptions of our worldviews and mental models in communities. This includes a need to *trigger conversations on learning*, following appropriate models for sustainability (such as The Doughnut, the United Nations Sustainable Development Goals, and the Principles for a Circular Economy), and finding *common ground* by *bridging the value(s)-action gap*. This phenomenon, identified in this paper, is characterised by the gap between intentions (represented by purposes, meanings, and values) and actual behaviour, which creates sustainable value with its broader impact (e.g., societal, environmental). Finding common ground between and with various stakeholders through community learning processes should lead to better alignment that puts *values into meaningful action*.

We suggest that the approach Community OR took in the late 1970 s, 1980 s, and beyond to emphasise a *meaningful engagement of communities* can also involve reflecting on marginalised issues of absent stakeholders, such as *nature* and *future generations*. This can contribute to the call for a shift in engaging higher levels of systems consciousness, accounting for the diverse needs of different stakeholders, ranging from *ego to ecosystem awareness* and reflection on *intergenerational needs and consequences* as part of community learning. This requires shaping worldviews that reflect our sustainability challenges, which is important in exploring the real-world context of a problem situation and the conceptual models based on a declared worldview. This goes to the heart of the *legitimacy* of a system that is *interdependent* upon *meeting the needs of people in harmony with living systems*. To address the sustainability challenge, two critical boundaries need to be considered: not *overshooting* our *ecological ceiling* (environmental impact) and *undershooting* our *human well-being* (societal impact). SSM is well-positioned to explore messy and complex sustainability challenges (the application area), the interconnectivity between the United Nations Sustainable Development Goals (referred to as ‘ideals’ in this paper), the purposes, meanings, and values of different individuals, organisations, and communities, and their actions, taken alongside, new meaning and learning gathered by engaging with appropriate models for sustainability.

Further avenues for research and practice are suggested to explore shaping worldviews and mental models for often marginalised, overlooked or even ignored issues and concerns of broader human interests, such as those presented by the sustainability challenge. The application of SSM (as well as other problem structuring methods) to highlight and bridge the perceived *value(s)-action gap* through triggering community-based double-loop learning should be further investigated. This can contribute to shifting mindsets and actions towards decarbonisation and a more circular, regenerative future.

## Data Availability

No datasets were generated or analysed during the current study.
